# Real world outcomes of intravitreal and systemic therapy in primary and secondary vitreoretinal lymphoma

**DOI:** 10.1038/s41598-026-37804-4

**Published:** 2026-02-14

**Authors:** Sina A. Beer, Carola Huber, Emil Nasyrov, Lasse Wolfram, Martin Pietzsch, Aileen Schenk, Michael Sommer, Andreas Wedrich, Claudia Lengerke, Deshka Doycheva, Christoph Deuter, Daniela Süsskind, Stefan Wirths, David A. Merle

**Affiliations:** 1 Department of Internal Medicine II, Hematology, Oncology, Clinical Immunology and Rheumatology, University Hospital Tübingen, Tübingen, Germany; 2 Department of Ophthalmology, Medical University of Graz, Graz, Austria; 3 Centre for Ophthalmology, University Hospital Tübingen, Tübingen, Germany

**Keywords:** Vitreoretinal lymphoma, Orphan disease, Relapse-free survival, PVRL, PIOL, Non-hodgkin lymphoma, Outcomes research

## Abstract

Vitreoretinal large B-cell lymphoma (VR-LBCL) is a rare hematologic malignancy. It is classified as primary (PVR-LBCL) or secondary (SVR-LBCL) based on the initial site of manifestation. Owing to limited prospective data and absent standardized guidelines, treatment remains challenging. This dual-center retrospective study aimed to evaluate outcomes of methotrexate (MTX) intravitreal (itv.), Rituximab itv., or combinatorial itv. therapy (R-MTX) and assess the impact of additional systemic immunochemotherapy. Among 65 patients (median age 72 years) included, 55.4% (*n* = 36) had PVR-LBCL. The median time to diagnosis was 31 days (1–2805). Over a median follow-up of 23.2 months, 35 patients relapsed. MTX itv. showed a trend toward better ocular relapse-free-survival than Rituximab itv. (*P* = 0.07). Intriguingly, patients receiving R-MTX itv. experienced no relapses throughout follow-up. In general, addition of systemic therapy was associated with significantly longer relapse-free-survival compared to itv. monotherapy (*P* = 0.05). Keratopathy and elevated intraocular pressure were common side effects with MTX itv. and Rituximab itv., respectively. Despite compelling findings and being one of the largest cohorts published to date, the heterogeneity of the patient population and small subgroups limit direct clinical translation. Nonetheless, this study provides a strong foundation for the design of future clinical trials.

**Supplementary Information:**

The online version contains supplementary material available at 10.1038/s41598-026-37804-4.

## Introduction

Vitreoretinal large B-cell lymphoma (VR-LBCL) is a rare and aggressive type of diffuse large B-cell lymphoma originating in the intraocular compartment, predominantly affecting the retina and vitreous^1^. It is classified as primary or secondary based on the initial site of manifestation and is recognized as an orphan disease (ORPHA:279904)^2^. Primary VR-LBCL (PVR-LBCL) is defined by exclusive initial ocular manifestation and categorized under primary large B-cell lymphoma of immune-privileged sites (IP-LBCL) in the 2024 WHO classification. Secondary VR-LBCL (SVR-LBCL) develops following a diagnosis of lymphoma outside the eye, such as primary central nervous system lymphoma (PCNSL) or systemic large B-cell lymphoma^3^. VR-LBCL patients with simultaneous PCNSL (15%) are also classified as SVR-LBCL^4^. A major concern with PVR-LBCL is the high rate of secondary central nervous system (CNS) involvement (56–90%), which significantly limits survival^1,5–7^. Progression-free survival (PFS) and overall survival (OS) for PVR-LBCL are reported at 18–31 months^8,9^ and 58–75 months, respectively^5,8^.

Clinically, PVR-LBCL often mimics uveitis, a phenomenon known as masquerade syndrome, presenting with nonspecific symptoms such as blurred vision and floaters and potentially showing a temporary response to corticosteroid treatment. This results in diagnostic delays, with reports ranging from 6 to 40 months from symptom onset to diagnosis^5^, hindering the timely initiation of effective treatment strategies. Furthermore, PVR-LBCL treatment remains challenging due to the lack of standardized protocols, leading to individualized approaches including intravitreal (itv.) therapy, radiation, and systemic chemo-immunotherapy, but with limited guidance on optimal combinations or sequencing^10^. Regarding itv. therapy, Methotrexate (MTX) is commonly referenced, with well-defined dosing intervals and dosages^11–13^. Still, MTX itv. is associated with notable ocular complications, including the development of cataract, corneal damage, and MTX-related maculopathy^14^. Alternatively, Rituximab itv. is more commonly used for patient’s refractory to MTX itv., however, it lacks standardized intervals and dosages. Smaller studies reported favorable outcomes for Rituximab itv. as a first-line treatment, although a tendency for earlier relapse was observed compared to MTX itv^15–19^. The combination of Rituximab and MTX itv. (R-MTX itv.) has only been described in a few case reports to date^20,21^.

While itv. treatment effectively clears intraocular tumor cells, evidence does not clearly support its role in preventing CNS relapse and it mostly fails to achieve long-term local control^1^. In this context, adding systemic treatment, particularly high-dose (HD) MTX, has proven effective in reducing the risk of CNS relapse^22^. Alongside HD-MTX, commonly used systemic agents include Rituximab, procarbazine, cytarabine, and vincristine^20,23^. In the relapse setting, high-dose chemotherapy followed by autologous stem cell transplantation (HDC/ASCT) also plays a growing role^1^. Some evidence supports the use of additional systemic treatment upfront in patients with bilateral eye involvement^20^.

Overall, due to the absence of definitive guidelines, the choice of therapy largely depends on patient characteristics and, most importantly, on the institutional protocol. In order to provide a better comparison of itv. treatments, this retrospective analysis aims to compare MTX itv. with Rituximab itv. across two centers that primarily use one of these agents in ocular first-line therapy.

## Results

### Study cohort (*n* = 65)

**Fig. 1:**
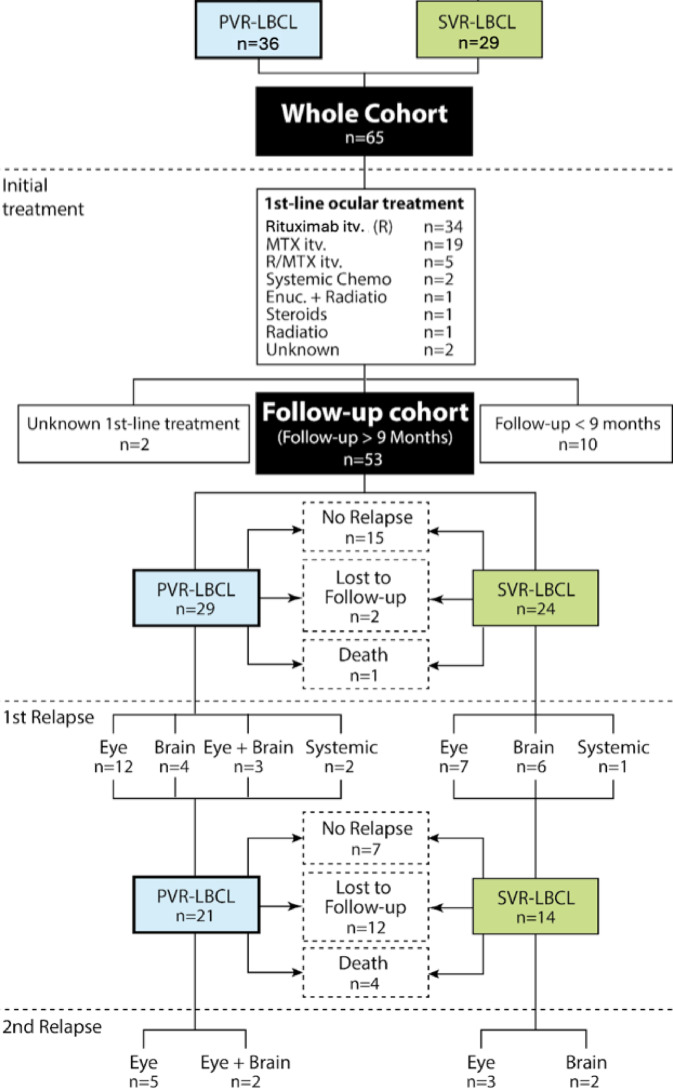
Study flow and treatment overview in VR-LBCL patients. Study flow of *n* = 65 VR-LBCL patients (corresponding to 99 eyes) from Tübingen and Graz, including initial treatments, and follow-up cohort composition.

A total of 65 patients diagnosed with either PVR-LBCL or SVR-LBCL were identified, including 34 patients from Tübingen and 31 from Graz. PVR-LBCL represented 55.4% (*n* = 36) of cases, while SVR-LBCL accounted for 44.6% (*n* = 29). VR-LBCL diagnosis via pars plana vitrectomy (PPV) was performed in 80% of cases (*n* = 52, Table 1), including four cases with concurrent retinal/choroidal biopsies. The other 20%, all of which SVR-LBCL, were diagnosed through biopsies of other tissues, such as skin, brain, or mammary tissue. All SVR-LBCL cases received systemic treatment before ocular lymphoma diagnosis. The median age at diagnosis across the entire cohort was 72.2 years (range: 49 to 96), and the median follow-up period was 23.2 months (range: 2.3 to 260.0). Detailed demographic and clinical characteristics of the cohort are summarized in Table 1 with center-specific data available in Table S1. The study flow is illustrated in Fig. 1.

**Table 1:** Characteristics, diagnostic methods, and first-line line intravitreal (itv.) treatment of the study cohort (*n* = 65).

Study cohort	Information
Study period	2000–2024
Study participants (Tübingen / Graz)	*n* = 65 (34 / 31)
Age at diagnosis, mean (range)	72.7 (49–96)
Sex, women	55.4% (*n* = 36)
Rheumatologic disease	*n* = 1 (limited scleroderma)
Virological assessment, serological detection	
HIV infection	None (*n* = 35 unknown)
Other infection (active / past)	None / *n* = 7 (Hepatitis B, B. burgdorferi, HSV-1)
Primary VR-LBCL (PVR-LBCL)	55.4% (*n* = 36)
Secondary VR-LBCL (SVR-LBCL)	44.6% (*n* = 29)
Sequence: (1) following primary systemic DLBCL / PCNSL; (2) concurrent manifestation (VRL + PCNSL / syst. DLBCL / Meningeosis), (3) unknown	(1) *n* = 11 / *n* = 9; (2) *n* = 4 / *n* = 2 / *n* = 2; (3) *n* = 1*
Systemic therapy at VRL diagnosis	None
Disease status at VRL diagnosis	CR: 13; PR: 1; SD: 1; Unknown: 1
Interval from completion of systemic therapy to VRL diagnosis, months	Median: 20 (range 5-233)

Diagnostics	Information
Initial ophthalmologic diagnosis considered VR-LBCL as differential diagnosis	70.8% (*n* = 46)
Ophtal. treatment before VR-LBCL diagnosis (topical / systemic: antiviral or steroids / unknown)	*n* = 44 / *n* = 13 / *n* = 4
Lymphoma laterality (mono- / binocular / unknown)	*n* = 29 / *n* = 34 / *n* = 2
Time to diagnosis (days), median (range)	31 (1–2805)
Diagnostic modality, PPV	80% (*n* = 52)
CSF	67.7% (*n* = 44)
cMRI	86.2% (*n* = 56)
FACS	49.2% (*n* = 32)
Histology	83.0% (*n* = 54)

1st line itv. treatment	Information
R / MTX / R-MTX / Other / Unknown	52.3% (*n* = 34) / 29.2% (*n* = 19) / 7.7% (*n* = 5) / 7.7% (*n* = 5) / 3% (*n* = 2)
Injections/eye, median (range)	5 (1–34)

PVR-LBCL = primary vitreoretinal lymphoma, SVR-LBCL = secondary vitreoretinal lymphoma, DLBCL = diffuse large B cell lymphoma, PCNSL = primary central nervous system lymphoma, CR = Complete response, PR = partial response, SD = stable disease, PPV = pars plana vitrectomy, CSF: Cerebrospinal Fluid, cMRI: cranial magnetic resonance imaging, MTX = methotrexate, R = Rituximab.

*No staging was performed due to the decision to pursue a palliative approach.

During initial ophthalmic evaluation, VR-LBCL was considered as a differential diagnosis in 70.8% (*n* = 46) of cases, with a median time to diagnosis of 31 days (range: 1 to 2805 days). Before the definitive lymphoma diagnosis, 67.7% (*n* = 44) of patients received topical ocular therapy, including anti-inflammatory agents (e.g., prednisolone acetate, dexamethasone), immunosuppressants (e.g., ciclosporin, mycophenolate mofetil), antibiotics (e.g., moxifloxacin, gentamicin), or lubricating eye drops. Systemic antiviral or glucocorticoid treatments were administered to 20% of patients.

Ocular first-line treatments included Rituximab itv. (52.3%, *n* = 34), MTX itv. (29.2%, *n* = 19), or R-MTX itv. (7.7%, *n* = 5). The remaining 7.7% (*n* = 5) received systemic chemotherapy, local radiation, or enucleation, whereas for two patients (3.0%) no documentation of treatment was available (Fig. 1). For Rituximab itv., the first-line treatment compromised six itv. injections of Rituximab (1 mg/0.1 mL) administered 4 weeks apart. The staggered protocol for MTX itv., according to Frenkel et al. ^25^, consisted of 400 µg/0.1 ml MTX itv. twice weekly for 4 weeks, once weekly for 8 weeks, and once monthly for 9 months. R-MTX regimens followed no defined protocol and were given at the discretion of the treating physician. In this study, R-MTX was defined as the administration of both MTX and Rituximab intravitreally within the first six injections. In most cases, combination therapy resulted from the development of MTX-induced keratopathy, prompting a switch to Rituximab. All injections were administered separately, with no instance of concurrent MTX and Rituximab injection at the same visit. The interval between injections was at least one month in all cases. The dosages used for MTX and Rituximab in the R-MTX group followed the respective monotherapy protocols. Despite these guidelines, many patients did not receive the full course of injections, primarily due to perceived individual treatment burden or a transition to systemic therapy regimens. Across all itv. treatments, a median of 5 (range: 1 to 34) injections were administered until local remission was achieved. Patients treated with Rituximab itv. received a median of 4 (range: 1 to 8) injections, whereas those treated with MTX itv. received a median of 7 (range: 1 to 34) injections.

### Follow-up cohort (*n* = 53)

#### Treatments

For subsequent analyses, only patients with a minimum follow-up period of 9 months were included, resulting in a total of 53 patients. The median follow-up was 34.1 months (range: 9 to 260), and the median age at diagnosis was 73 years (range: 49 to 96). Among these patients, 54.7% (*n* = 29) had PVR-LBCL, and 45.3% (*n* = 24) had SVR-LBCL (Fig. 1). Detailed follow-up cohort characteristics are presented in Table 2. Ocular first-line treatment in this cohort compromised Rituximab itv. (52.8%, *n* = 28), followed by MTX itv. (28.3%, *n* = 15), R-MTX itv. (9.4%, *n* = 5), and other regimens (9.4%, *n* = 5).

**Table 2:** Characteristics, first-line line intravitreal (itv.) treatment, additional systemic therapies, side effects and outcomes of the follow-up cohort (*n* = 53).

Follow-up cohort	Information
Follow-up (months), median (range)	34.1 (9–260)
Age at diagnosis, mean (range, median)	71.2 (49–96, 73)
Sex, women	52.8% (*n* = 28)
PVR-LBCL	54.7% (*n* = 29)
SVR-LBCL	45.3% (*n* = 24)

1st line itv. Treatment	Information
VR-LBCL: R / MTX / R-MTX / Other	52.8% (*n* = 28) / 28.3% (*n* = 15) / 9.4% (*n* = 5) / 9.4% (*n* = 5)
PVR-LBCL: R / MTX / R-MTX / Other	48.2% (*n* = 14) / 31.0% (*n* = 9) / 13.8% (*n* = 4) / 6.9% (*n* = 2)
SVR-LBCL: R / MTX / R-MTX / Other	58.3% (*n* = 14) / 25.0% (*n* = 6) / 4.2% (*n* = 1) / 12.5% (*n* = 3)

Systemic treatment	Information
Systemic treatment, yes / no	85% (*n* = 45) / 15% (*n* = 8)
Time point of systemic treatment	
Before VR-LBCL diagnosis (all SVR-LBCL)	30.2% (*n* = 16)
Additional to 1st line itv. treatment	39.6% (*n* = 21: *n* = 13 PVR-; *n* = 8 SVR-LBCL)
Within the VRL treatment course	15% (*n* = 8)
Type of systemic treatment	
Chemo-immunotherapy	*n* = 38
Other (Radiatio, i.th. Therapy)	*n* = 7
Additional HDC/ASCT 1st / 2nd line	*n* = 9 / *n* = 4

Side effects	Information
Keratopathy after 1st line (all MTX itv. associated)	*n* = 11 (55% of MTX-treated patients)
IOP decompensation after 1st line (all Rituximab itv. associated)	*n* = 13 (39.4% of Rituximab-treated patients)
Secondary malignancies (besides DLBCL axis)	*n* = 2 (Mamma-Ca, AML)

Final outcome	Information
Alive	*n* = 19
Death / Palliation	*n* = 5 / *n* = 11
Lost to follow-up (by 1st / 2nd relapse evaluation / later)	*n* = 18 (2 / 12 / 4)
Course of disease, including prior history & relapses	
Exclusive vitreoretinal manifestation	32.0% (*n* = 17)
Other than vitreoretinal manifestation (systemic, CNS) / unknown	62.3% (*n* = 33) / 5.6% (*n* = 3)

PVR-LBCL = primary vitreoretinal lymphoma, SVR-LBCL = secondary vitreoretinal lymphoma, i.th. = intrathecal, DLBCL = diffuse large B cell lymphoma, CNS = central nervous system, MTX = methotrexate, R = rituximab.

66.0% (*n* = 35) of patients experienced a relapse after first-line VR-LBCL treatment and received second-line therapy. Second-line ocular treatment included Rituximab itv. (31.4%, *n* = 11), local radiation therapy (11.4%, *n* = 4), R-MTX itv. (5.7%, *n* = 2) and MTX itv. (2.9%, *n* = 1). 14.3% (*n* = 5) of relapsed patients received systemic therapy, while 2.9% (*n* = 1) transitioned to palliative care. Second-line treatment was undocumented for 31.4% (*n* = 11), primarily due to transfers to other centers.

At any point during the entire treatment course, systemic therapy was administered to a total of 85.0% (*n* = 45), including 30.2% as pre-VRL-LBCL diagnosis treatment (all corresponding to SVR-LBCL), 39.6% concurrent with first-line ocular therapy, and 15.0% in subsequent treatment phases (Table 2). Of the 39.6% (*n* = 21) who received systemic therapy in addition to first-line ocular treatment, of which 13 were PVR-LBCL and eight were SVR-LBCL. Chemo-immunotherapy was the most common systemic approach (84.4%, *n* = 38), with 71.1% (*n* = 27) Rituximab-based and 28.9% (*n* = 11) MTX-based regimens. 13.2% of patients received either systemic radiation or intrathecal therapy alone. HDC/ASCT was used in 17.0% (*n* = 9) of patients during first-line therapy and 7.5% (*n* = 4) during second-line therapy (Table 2).

#### Clinical outcomes

Among 35 patients with relapse following first-line VR-LBCL therapy, 54.3% (*n* = 19) had isolated, eye-confined relapse, while 45.7% (*n* = 16) experienced non-eye-confined relapse involving the brain, simultaneous eye and brain, or systemic sites. The median time to relapse at any site was 8.9 months (range: 2.9 to 129.6). No statistically significant difference in time to relapse between eye-confined and non-eye-confined relapses was found (Table 3). Missing data precluded reliable statistical analyses beyond the initial relapse. The following results focus on the PVR-LBCL group (*n* = 29), while also encompassing data from the entire VR-LBCL follow-up cohort (*n* = 53) and analyzing the impact of age and sex on relapse characteristics.

**Table 3:** Information on relapses, as well as second-line treatments of the study cohort (*n* = 53).

1st relapse	Information
No relapse / unknown	*n* = 15 / 3
Confirmed relapse (any manifestation)	66.0% (*n* = 35)
PVR-LBCL	72.4% (*n* = 21)
SVR-LBCL	58.3% (*n* = 14)
Relapse manifestation: eye / brain / eye + brain / systemic	*n* = 19 / *n* = 10 / *n* = 3 / *n* = 3
Time to 1st relapse (months), median (range, average)	
Relapse at any site	8.9 (2.9–129.6, 20.6)
Relapse eye	8.3 (4.1–126.2, 16.9)
Relapse other than eye	8.9 (2.9–129.6, 27.0)

2nd line treatment	Information
Number of patients with 2nd line treatment	*n* = 35
R / Radiatio / R-MTX / Radiatio + MTX	*n* = 11 / *n* = 4 / *n* = 2 / *n* = 1
itv. + immunotherapy / immunotherapy only	*n* = 3 / *n* = 2
Palliative / unknown	*n* = 1 / *n* = 11
2nd line injections/eye, median (range)	Not enough data to draw meaningful conclusions
2nd line treatment response vitreoretinal	
CR / PR	*n* = 11 / 4
Unknown	*n* = 20

2nd relapse	Information
Number of patients examinable for 2nd relapse	*n* = 19
Confirmed relapse (any manifestation)	63.2% (*n* = 12)
Relapse manifestation (eye / brain / eye + brain / systemic)	*n* = 3 / *n* = 6 / *n* = 2 / *n* = 1
Time to 2nd relapse (months), median (range, average)	*inconsistent documented*

PVR-LBCL = primary vitreoretinal lymphoma, SVR-LBCL = secondary vitreoretinal lymphoma, i.th. = intrathecal, DLBCL = diffuse large B cell lymphoma, CNS = central nervous system, MTX = methotrexate, R = rituximab.

#### Relapse-free survival

We conducted survival analyses to evaluate local and systemic disease control of different treatment regimens in patients with PVR-LBCL who relapsed either (a) at any site (non-eye-confined) or (b) in the eye only (eye-confined). We first evaluated intravitreal therapy alone, then assessed its combined potential with systemic therapy. Notably, none of the PVR-LBCL patients received systemic therapy before diagnosis.

##### Rituximab itv., MTX itv. and R-MTX itv. in PVR-LBCL: MTX and R-MTX itv. show a trend toward better local disease control

**Fig. 2:**
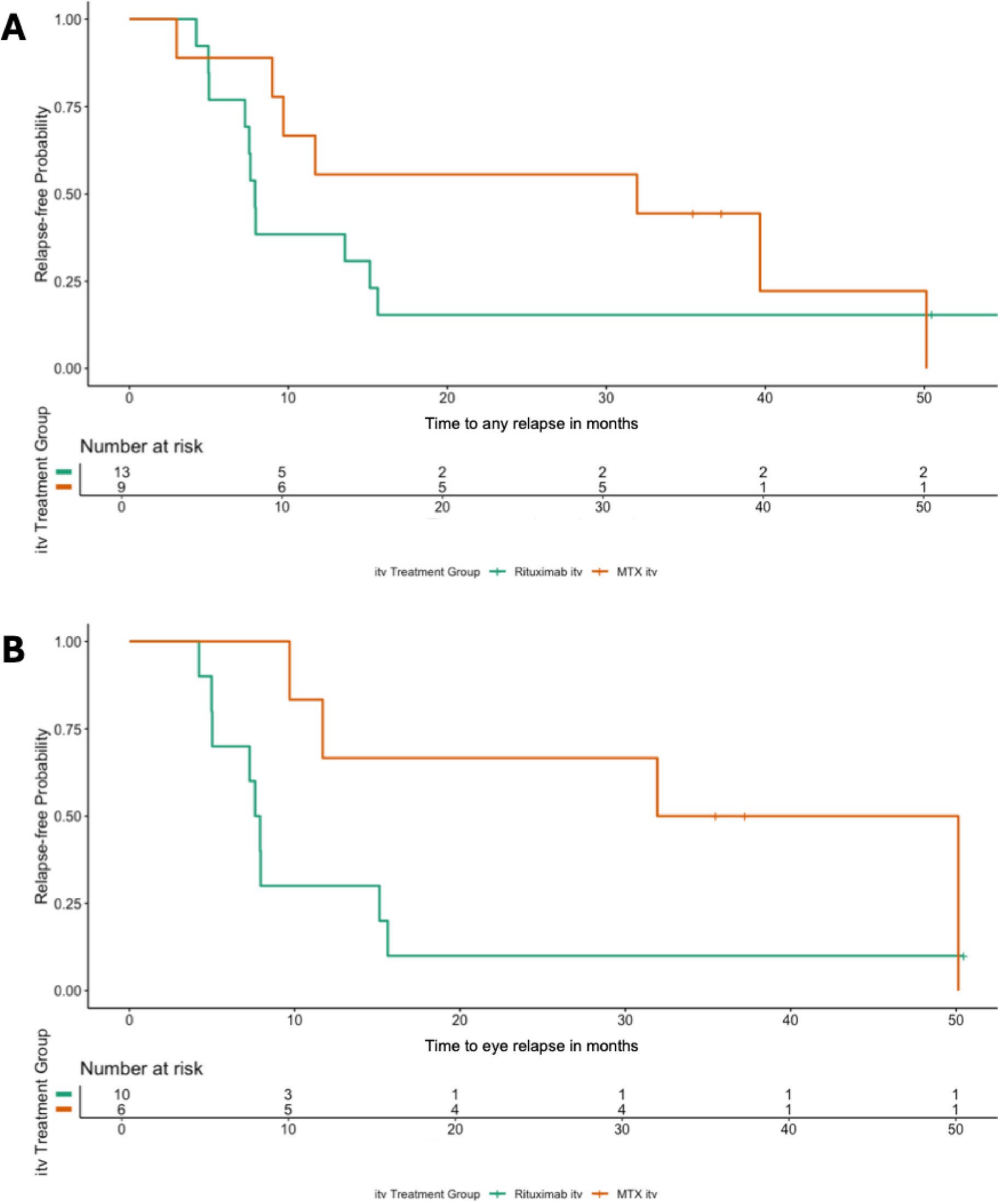
Relapse-free survival in PVR-LBCL patients by intravitreal therapy. (**A**) Relapse-free survival for any site relapse among 22 PVR-LBCL patients showed no significant difference between those treated with Rituximab intravitreal (itv., *n* = 13) and Methotrexate (MTX) itv. (*n* = 9) (*P* = 0.3). (**B**) Relapse-free survival for eye-confined relapses among 16 PVR-LBCL patients indicated a trend toward improved relapse-free survival with MTX itv. (*n* = 6) compared to Rituximab itv. (*n* = 10), though this difference was not statistically significant (*P* = 0.07).

Survival analyses were performed to compare relapse-free survival among patients with PVR-LBCL treated with Rituximab itv. (*n* = 13), MTX itv. (*n* = 9), or R-MTX itv. (*n* = 4). Systemic therapy administration was not taken into account in these analyses, although groups were well balanced (50% received additional systemic therapy, 50% did not). Comparing Rituximab and MTX itv. regimens, 22 PVR-LBCL patients were available for any site relapse analysis. There was no significant difference in relapse-free survival between patients treated with Rituximab itv. (*n* = 13) and those treated with MTX itv. (*n* = 9) (*P* = 0.3; Fig. 2a). For 16 PVR-LBCL patients with eye-confined relapses, a trend toward improved relapse-free survival with MTX itv. (*n* = 6) compared to Rituximab itv. (*n* = 10) was noted but did not reach statistical significance (*P* = 0.07; Fig. 2b). For patients who received R-MTX itv. (*n* = 4), a significant difference in relapse-free survival for both any site (*P* = 0.02; Figure S1a) and eye-confined relapses (*P* = 0.005; Figure S1b) was observed. Pairwise comparisons of any site relapses showed no significant difference between Rituximab itv. and MTX itv. (*P* = 0.31) but demonstrated superior outcomes with R-MTX itv. compared to both (*P* = 0.019 for each). In the eye-confined relapse cohort, R-MTX itv. was also associated with significantly longer relapse-free survival compared to Rituximab itv. (*P* = 0.021).

##### Intravitreal +/- systemic therapy in PVR-LBCL: combined intravitreal and systemic therapy demonstrates potential for improved relapse-free survival

**Fig. 3:**
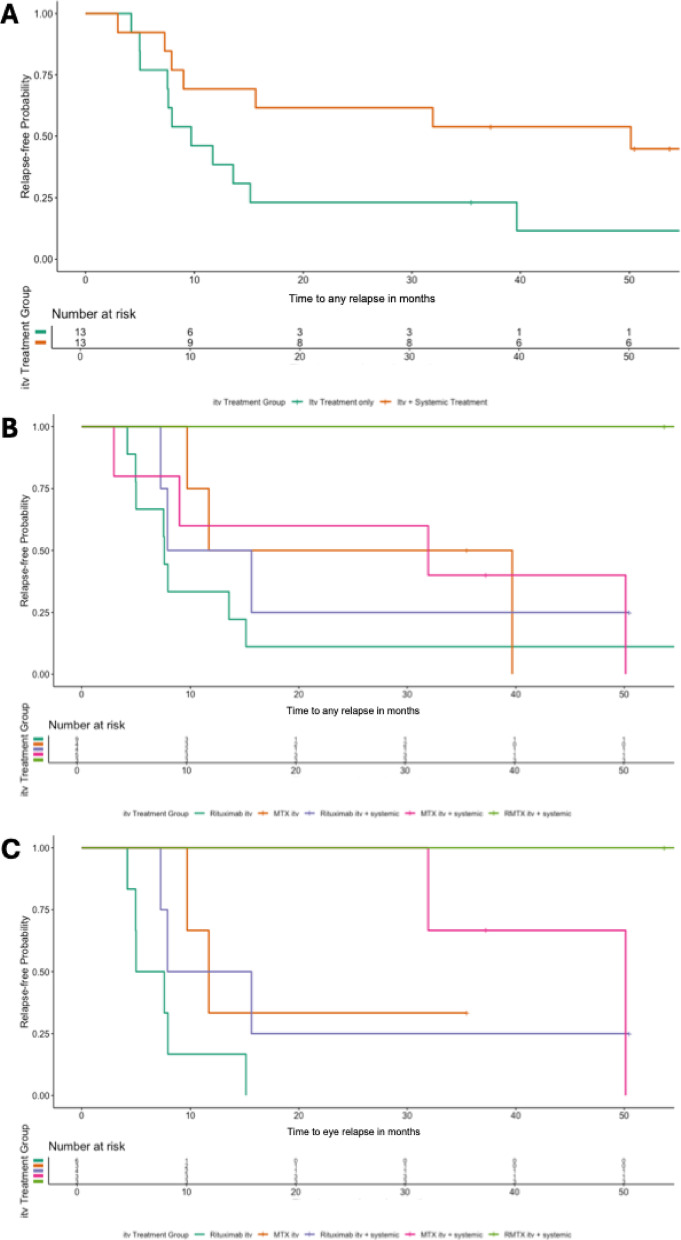
Relapse-free survival by combined intravitreal and systemic therapies in PVR-LBCL. (**A**) For any site relapses, patients receiving both systemic and intravitreal (itv.) therapy (*n* = 13) demonstrated significantly longer relapse-free survival compared to those treated with itv. monotherapy (*n* = 13) (*P* = 0.05). (**B**) Among different itv. treatment regimens combined with systemic therapy, the Rituximab-Methotrexate (R-MTX) itv. + systemic therapy group (*n* = 3) showed the most favorable outcomes with no observed relapses, though overall differences did not reach statistical significance (*P* = 0.1). (**C**) For eye-confined relapses, significant differences were observed across treatment groups (*P* = 0.003), with longer relapse-free survival in patients treated with MTX itv. + systemic therapy (*n* = 3) and R-MTX itv. + systemic therapy (*n* = 3) compared to other regimens. However, these differences were not significant after adjustment for multiple comparisons (adjusted *P* = 0.055).

To assess the impact of adding systemic immunochemotherapy to itv. therapy, we compared relapse-free survival outcomes between patients who received itv. therapy only (*n* = 13) and those who received itv. and systemic therapy in parallel (*n* = 13). Systemic immunochemotherapy, as described above, was predominantly Rituximab- and MTX-based: HD-MTX (*n* = 5), HDC/ASCT (*n* = 3), R-MTX (*n* = 2), R-AraC (*n* = 1), AraC intrathecal (*n* = 1), and radiation therapy (*n* = 1). For relapses at any site, patients receiving both itv. and systemic therapy had a significantly longer relapse-free survival compared to those receiving itv. therapy alone (*P* = 0.05; Fig. 3a). Notably, patients receiving both itv. and systemic treatment were significantly younger (mean age: 68.8 years) compared to those receiving itv. treatment only (mean age: 75.9 years, *P* = 0.025).

Next, we analyzed the added benefit of systemic treatments to the individual ocular treatment regimens. While overall differences among the different treatment regimens did not reach statistical significance (*P* = 0.1), the R-MTX itv. + systemic therapy group (*n* = 3) showed the most promising outcomes, with no observed relapses (Fig. 3b). For eye-confined relapses, significant differences in relapse-free survival were observed across treatment groups (*P* = 0.003; Fig. 3c). Patients treated with MTX itv. + systemic therapy (*n* = 3) and R-MTX itv. + systemic therapy (*n* = 3) tended to have longer relapse-free survival compared to those receiving Rituximab itv. alone (*n* = 6), MTX itv. alone (*n* = 3), or Rituximab itv. + systemic therapy (*n* = 4). Although these comparisons showed a trend toward improved outcomes, the differences did not reach statistical significance after adjustment for multiple comparisons (adjusted *P* = 0.055) and should be interpreted with caution given the small sample sizes.

##### PVR-LBCL vs. SVR-LBCL: Relapse-free survival shows no significant differences by VR-LBCL type

**Fig. 4:**
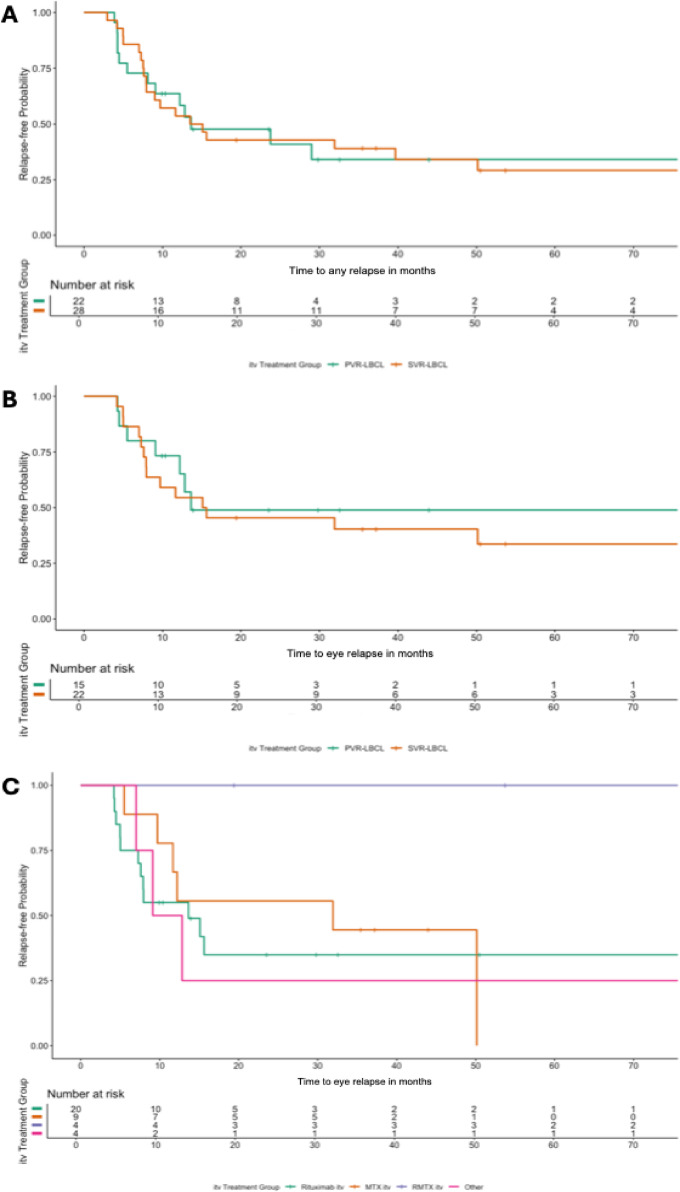
Relapse-Free survival in PVR-LBCL and SVR-LBCL patients and treatment effects in VR-LBCL. (**A**) Relapse-free survival for any site relapses showed no significant difference between PVR-LBCL (*n* = 28) and SVR-LBCL (*n* = 22) patients (*P* = 0.8). (**B**) For eye-confined relapses, no significant difference in relapse-free survival was observed between PVR-LBCL (*n* = 22) and SVR-LBCL (*n* = 15) patients (*P* = 0.7). (**C**) Relapse-free survival for eye-confined relapses across all VR-LBCL patients (*n* = 50) showed no significant differences among intravitreal (itv.) treatments (*P* = 0.2), though a trend toward improved outcomes with R-MTX itv. was observed.

We compared relapse-free survival between PVR- and SVR-LBCL patients. No significant differences in time to any site relapse were observed between the PVR-LBCL (*n* = 28) and SVR-LBCL (*n* = 22) (*P* = 0.8; Fig. 4a). Similarly, for eye-confined relapses, no significant differences were found between the two groups (PVR-LBCL *n* = 22; SVR-LBCL *n* = 15) (*P* = 0.7; Fig. 4b). In addition, no significant differences in eye-confined relapses were observed across the different itv. treatments (*P* = 0.2; Fig. 4c), with the caveat that 40% of patients also received concurrent systemic therapy.

Age and sex show no correlation with treatment regimen or relapse-free survival

##### Age and sex show no correlation with treatment regimen or relapse-free surviv

As patients age is a known risk factor for adverse outcomes in VR-LBCL, we evaluated the demographic characteristics of patients treated with MTX itv. and Rituximab itv. No significant differences were found in age (*P* = 0.19) or sex (*P* = 0.22) between the two treatment groups. To assess the impact of age on time to relapse, patients were divided into two groups based on the median age of 73 years (*n* = 26 < 73 years, *n* = 27 ≥ 73 years). No statistically significant difference in time to relapse was observed between the age groups for either VR-LBCL (*P* = 0.5) or PVR-LBCL (*P* = 0.2).

#### Ocular outcomes

To evaluate functional ophthalmic outcomes, BCVA was analyzed at three time points: (a) initial consultation at the treating eye clinic, (b) VR-LBCL diagnosis, and (c) after completion of first-line treatment. Subsequent time points were not analyzed due to missing data. All BCVA values are reported in logMAR.

##### BCVA shows no significant changes across time points or treatments

For VR-LBCL (*n* = 53), mean BCVA was 0.54 (range: -0.1 to 2.7) at initial consultation, 0.62 (range: -0.1 to 2.4) at diagnosis, and 0.63 (range: -0.1 to 3.0) after first-line treatment. No significant changes in BCVA were observed between successive time points (*P* = 0.90 and *P* = 0.85) or between initial consultation and after first-line treatment (*P* = 0.84). BCVA outcomes did not differ significantly between patients treated with Rituximab itv. or MTX itv. (*P* = 0.55). An additional linear mixed model analysis assessed the effects of treatment type (Rituximab itv. and MTX itv.) and time point on BCVA. The model revealed no significant main effects for time point (*P* = 0.70) or treatment type (*P* = 0.55). The interaction effect between time points and treatment was also not significant (*P* = 0.72).

##### Ocular side effects: keratopathy and IOP decompensation as treatment-associated complications

11 (55%) of 20 patients that received MTX itv. developed keratopathy (corneal epitheliopathy), while two patients who received Rituximab itv. had pre-existing keratopathy that showed no worsening under treatment. 13 (39.4%) of 33 patients who received Rituximab itv. experienced transient intraocular pressure elevation throughout the follow-up period. These were typically short-lived, with normalization or return to tolerable levels within several days. While peak IOP values reached up to 40 mmHg, the majority remained moderate (< 30 mmHg). Management strategies were heterogeneous and included topical and systemic antiglaucomatous agents, cyclophotocoagulation in rare cases, and paracentesis following injection in patients with borderline baseline IOP. In this subgroup, IOP remained well controlled without further intervention. Notably, no cases of sustained or uncontrolled IOP elevation directly attributable to Rituximab were observed. A more detailed analysis was limited by the heterogeneity of the cohort and the frequent presence of previous episodes of IOP elevation. Therefore, for this study, only IOP elevations in patients without elevated IOP at the time of diagnosis were considered for descriptive evaluation. No other itv. treatment-associated side-effects were documented.

## Discussion

The clinical management of VR-LBCL remains challenging due to its rarity and the lack of standardized treatment guidelines. This study highlighted significant heterogeneity in ocular treatment strategies, particularly in their combination with systemic therapeutic interventions, while also pointing out trends for potentially favorable regimes.

Ocular therapy for PVR-LBCL most commonly involves MTX or Rituximab itv., with a few reports on a very limited number of patients describing combinatorial approaches utilizing both agents^18,20,21^. However, the differential therapeutic efficacy of these regimens remains underexplored. To address this, we analyzed data from two centers, each employing either MTX or Rituximab itv. as first-line treatment. Our findings suggest that MTX itv. may offer superior ocular relapse-free survival compared to Rituximab monotherapy, consistent with prior studies demonstrating MTX’s effectiveness in controlling eye-confined disease^14,25^, while reports on Rituximab itv. showed more frequent relapses^17^. Based on published treatment protocols and as replicated in this study, patients receiving Rituximab during first-line therapy require fewer injections compared to those treated with MTX. However, in the absence of controlled clinical trials, it remains unclear whether increasing the injection frequency of Rituximab would result in comparable outcomes. Notably, although limited by low patient numbers, none of the patients that received R-MTX itv. in our cohort experienced relapse during the follow-up period. Hypothetically, this finding suggests an additive or synergistic effect that has also been introduced previously^18,21,26,27^. Similar effects have been observed in PCNSL with combined systemic MTX and Rituximab therapy^28,29^, although this was not consistent across all studies^30^. A considerable confounding factor in these eye-confined relapse analyses is the inclusion of patients who received additional systemic treatment. However, the number of such patients well balanced (50% received additional systemic therapy, 50% did not) and limited (*n* = 5 in the Rituximab itv., *n* = 4 in the MTX itv group). Statistical analysis excluding all patients who received systemic therapy revealed a similar trend favoring MTX itv. although the results did not reach significance, most likely due to consequently low patient numbers.

Adding systemic therapy to intravitreal treatment significantly improved relapse-free survival in PVR-LBCL patients compared to intravitreal therapy alone. This finding aligns with a previous study demonstrating that systemic chemotherapy, while not preventing CNS involvement in PVR-LBCL, effectively delayed its onset^22^. Despite the significant difference in age between the two groups in our study, these insights point toward a potential benefit of a combined local and systemic therapeutic approach, especially when taking the high risk of CNS involvement into account. Interestingly, the 15 patients who have remained relapse-free to date exhibited no discernible demographic trends, yet 13 of 15 received systemic therapy during their treatment course. Of which five were PVR-LBCL patients and underwent combined itv. and frontline systemic therapy.

The relatively rare but observable extraocular and CNS manifestations underscore the importance of a comprehensive diagnostic workup, as emphasized in previous studies^31^. Additionally, integrating genetic data could enhance the prediction of CNS involvement risk and help to rationally guide treatment decisions in the future^32^.

From an ophthalmic perspective, intraocular lymphoma was frequently considered a differential diagnosis at the time of initial consultation in most cases across both centers, reflecting physicians’ awareness of the potential for masquerade syndrome. The diagnostic workup revealed notable differences between centers, with additional parameters such as the IL-10/IL-6 ratio not consistently reported^33^. In unclear cases, incorporating such analyses could be beneficial, as they have been shown to offer high discriminatory power for diagnosing VR-LBCL^34^. Intriguingly, BCVA remained stable across treatment groups, with no significant changes from initial consultation to post-treatment follow-up. Likewise, mean BCVA did not differ significantly between MTX itv. and Rituximab itv., suggesting that both agents preserve visual function. This aligns with previous reports that itv. chemotherapy controls ocular disease without compromising visual acuity^35^. However, this finding may be misleading, as the impact on BCVA largely depends on the site of intraocular manifestation. If the vitreous is affected, visual acuity depends on the density of cellular infiltration, which can vary significantly between patients and typically improves markedly after diagnostic vitrectomy. When the retina is involved, the location of retinal infiltrates is critical. Infiltrates in the macula are associated with poorer visual acuity and generally show no improvement following therapy. Due to insufficiently detailed information on intraocular manifestation across all patients, reliable subgroup analyses could not be performed. Observed rates of ocular side effects, including keratopathy for MTX itv. and intraocular pressure elevation for Rituximab itv., were similar to reported rates in published literature^17,36^.

This study is limited by its small sample size, particularly in the combination therapy group, which restricts statistical power and generalizability. Selection bias and inter-center treatment heterogeneity may also have impacted the findings. Additionally, missing data from patient transfers and incomplete documentation complicated analyses, especially for long-term outcomes beyond first relapse after VR-LBCL therapy. Despite these limitations, it is important to highlight that the analyzed cohort represents one of the largest to be published to date, with a sample size significantly larger than the seminal study on intravitreal MTX treatment^25^, which included 44 eyes from 26 patients. The study provides valuable insights into current VR-LBCL treatment practices and outcomes. Prospective randomized trials are needed to validate these findings and establish VR-LBCL management guidelines. Future research should compare MTX, Rituximab, and combination therapies, refine dosing, and explore concurrent systemic therapy as well as predictive biomarkers for personalized treatment.

In conclusion, MTX itv. may offer superior relapse-free survival over Rituximab itv., with itv. combination therapies and added systemic treatment showing promising results in disease control. Larger studies are required to confirm these results and standardize care.

## Methods

### Patient cohort and clinical data sources

This retrospective dual center study included all patients diagnosed with PVR-LBCL or SVR-LBCL at the University Hospital Tübingen, Germany, and the University Hospital Graz, Austria between 2000 and 2024. This study was approved and informed consent was waived, due to the retrospective nature of the study, by the Ethics Committee of the Medical Faculty of the University of Tübingen (reference no. 096/2023B02) and and by the Ethics Committee of the Medical Faculty of the University of Graz (reference no. 35–279 ex 22/23). Data were extracted from electronic medical records, including demographic characteristics, ophthalmic and hematologic examination findings, best-corrected visual acuity (BCVA), laboratory parameters, time to diagnosis, time to relapse, therapeutic interventions, therapy-induced complications, and clinical outcomes. Ocular remission was defined as the absence of detectable intraocular lymphoma cells on ophthalmic examination. Ocular relapse was defined as the recurrence of intraocular lymphoma activity in a previously treated eye, based on clinical findings (e.g., vitritis, subretinal infiltrates) and confirmed cytologically or radiologically where available.

All enrolled patients formed the study cohort, focusing on clinical presentation at first evaluation, time to diagnosis, distribution of PVR- vs. SVR-LBCL, and initiated first-line treatments. For robust outcome analysis, including relapses, only patients with a minimum follow-up of 9 months were included, referred to as the follow-up cohort (Fig. 1). To accurately distinguish PVR-LBCL from SVR-LBCL, we reviewed cerebral MRI, whole-body CT, histology reports, and flow cytometry results of blood, cerebrospinal fluid, and bone marrow, as accessible. Cytokine analyses (e.g., IL-6/IL-10 ratios from anterior chamber taps) were not routinely performed across centers and were therefore excluded from statistical analyses due to limited availability and lack of standardization.

### Statistical methods

Data collection, sharing, and tidying were performed using Microsoft Excel (Version 16.87). Statistical analyses were conducted in R-Studio (R Version 4.4.0, R-Studio Version 2024.04.2 + 764). The analysis was conducted at the patient level. In cases with bilateral ocular involvement at initial consultation, the right eye was defined as the study eye for all eye-specific analyses. Continuous variables were summarized as means ± standard deviation or median with interquartile ranges, as appropriate. Patients’ age, treated as a continuous variable, was compared between groups using the Wilcoxon rank sum test with continuity correction. Same applies for best-corrected visual acuity (BCVA) values between time points, which were converted to logMAR using the method described by Moussa et al.^24^. Additionally, a linear mixed model analysis was conducted to assess the effects of treatment type (Rituximab itv. and MTX itv.) and time point on BCVA.

Categorical variables, including binary outcomes, were presented as frequencies and percentages and analyzed using Fisher’s exact test or Chi-squared tests, as applicable. A two-sided p-value of < 0.05 was considered statistically significant for all analyses. Kaplan-Meier estimators were calculated using the *survminer* and *survival* packages in R. Survival curves were compared using the log-rank test. When the overall log-rank test (chi-squared statistic) indicated a significant difference, pairwise comparisons between treatment groups were performed. To account for multiple comparisons and control the false discovery rate, *P*-values were corrected using the Benjamini-Hochberg procedure. Patients with missing data were excluded from the analysis.

## Supplementary Information

Below is the link to the electronic supplementary material.

Supplementary Material 1

## Data Availability

The datasets generated and analyzed during the current study are available from the corresponding author upon reasonable request.
