# Supplementary material for: Real world outcomes of intravitreal and systemic therapy in primary and secondary vitreoretinal lymphoma
Source: Sci Rep. 2026 Feb 14;16:6513. doi: 10.1038/s41598-026-37804-4 (PMC12910012; doi:10.1038/s41598-026-37804-4)
Supplement: Supplementary file 1 — Supplementary Material 1 [file 41598_2026_37804_MOESM1_ESM.docx]

Supplementary Material

Figure S1:


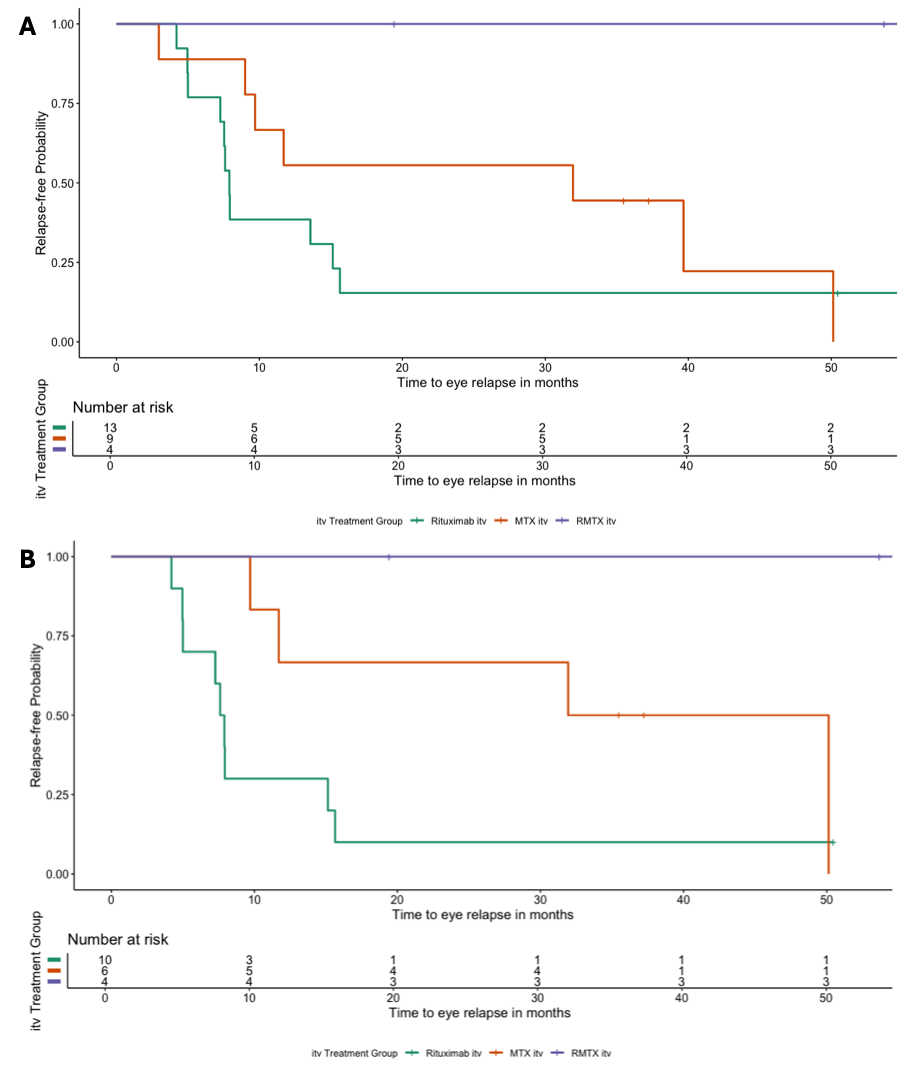


**Figure S1: Relapse-Free Survival Among PVR-LBCL Patients by Intravitreal Treatment including Rituximab-Methotrexate combination.** (A) For all-site relapses, a significant difference in relapse-free survival was observed among patients treated with Rituximab itv. (n=13), MTX itv. (n=9), and R-MTX itv. (n=4) (P = 0.02), with R-MTX itv. showing superior outcomes compared to both Rituximab itv. and MTX itv. (*P* = 0.019 for each). (B) For ocular-only relapses, significant differences were also observed among the three groups (*P* = 0.005), with R-MTX itv. associated with significantly longer survival compared to Rituximab itv. (P = 0.021). Comparisons between R-MTX itv. and MTX itv. (P = 0.052) and between Rituximab itv. and MTX itv. (P = 0.072) approached statistical significance, favoring R-MTX itv.

Table S1:

|  | **Tübingen** | **Graz** |
| --- | --- | --- |
| **Number of patients** | n=34 | n=31 |
| **Age, mean (range)** | 74.2 (49-86) | 71.1 (49-96) |
| **Sex, women** | 67.6% (n=23) | 41.9% (n=13) |
| **Lymphoma diagnosis, PVR-LBCL** | 50% (n=17) | 61.3% (n=19) |
| **Lymphoma diagnosis, SVR-LBCL** | 50% (n=17: systemic DLBCL n=7, PCNSL n=5, concurrent manifestation n=5) | 38.7% (n=8: systemic DLBCL n=4, PCNSL n=4, concurrent manifestation n=3,  unknown n=1) |
| **Follow-up study cohort, months, median (range)** | 15 (2-111) | 24 (1-259) |
| **Lost to follow-up study cohort, in total** | n=13 | n=20 |
| **Death (thereof, treatment-related mortality)** | n=3 (n=1) | n=2 (n=0) |
| **1st LINE TREATMENT, study cohort** |  |  |
| **Rituximab itv.** | 85.3% (n=29) | 16.1% (n=5) |
| **MTX itv.** | 2.9% (n=1) | 58.1% (n=18) |
| **Rituximab/MTX itv.** | none | 16.1% (n=5) |
| **Other** | Systemic CTx: 5.9% (n=2), Radiatio: 2.9% (n=1), unknown (n=1) | Steroids only (n=1), Radiatio (n=1), unknown (n=1) |
| **Injections/eye, median (range)** | 4 (1-19) | 7 (1-34) |

**Table S1: Center-specific characteristics of the study cohort.** PVR-LBCL = Primary Vitreoretinal Lymphoma, SVR-LBCL = Secondary Vitreoretinal Lymphoma, itv. = intravitreal, DLBCL = diffuse large B cell lymphoma, PCNSL = primary central nervous system lymphoma, CTx = chemotherapy, MTX = methotrexate.
